# Transcriptome sequencing in an ecologically important tree species: assembly, annotation, and marker discovery

**DOI:** 10.1186/1471-2164-11-180

**Published:** 2010-03-16

**Authors:** Thomas L Parchman, Katherine S Geist, Johan A Grahnen, Craig W Benkman, C Alex Buerkle

**Affiliations:** 1 Department of Botany, University of Wyoming, Laramie, WY 82071, USA; 2 Department of Biology, Beloit College, Beloit, WI 53511, USA; 3 Department of Molecular Biology, Laramie, WY 82071, USA; 4 Department of Zoology and Physiology, University of Wyoming, Laramie, WY 82071, USA

## Abstract

**Background:**

Massively parallel sequencing of cDNA is now an efficient route for generating enormous sequence collections that represent expressed genes. This approach provides a valuable starting point for characterizing functional genetic variation in non-model organisms, especially where whole genome sequencing efforts are currently cost and time prohibitive. The large and complex genomes of pines (*Pinus *spp.) have hindered the development of genomic resources, despite the ecological and economical importance of the group. While most genomic studies have focused on a single species (*P. taeda*), genomic level resources for other pines are insufficiently developed to facilitate ecological genomic research. Lodgepole pine (*P. contorta*) is an ecologically important foundation species of montane forest ecosystems and exhibits substantial adaptive variation across its range in western North America. Here we describe a sequencing study of expressed genes from *P. contorta*, including their assembly and annotation, and their potential for molecular marker development to support population and association genetic studies.

**Results:**

We obtained 586,732 sequencing reads from a 454 GS XLR70 Titanium pyrosequencer (mean length: 306 base pairs). A combination of reference-based and *de novo *assemblies yielded 63,657 contigs, with 239,793 reads remaining as singletons. Based on sequence similarity with known proteins, these sequences represent approximately 17,000 unique genes, many of which are well covered by contig sequences. This sequence collection also included a surprisingly large number of retrotransposon sequences, suggesting that they are highly transcriptionally active in the tissues we sampled. We located and characterized thousands of simple sequence repeats and single nucleotide polymorphisms as potential molecular markers in our assembled and annotated sequences. High quality PCR primers were designed for a substantial number of the SSR loci, and a large number of these were amplified successfully in initial screening.

**Conclusions:**

This sequence collection represents a major genomic resource for *P. contorta*, and the large number of genetic markers characterized should contribute to future research in this and other pines. Our results illustrate the utility of next generation sequencing as a basis for marker development and population genomics in non-model species.

## Background

Large numbers of molecular markers and sequence data from across the genome are playing an increasingly important role in population genomic studies of fine-scale genetic variation and the genetic basis of traits [1]. Nevertheless, we lack genomic resources for most non-model organisms and whole genome sequencing is still largely impractical for most eukaryotes. Transcriptome, or Expressed Sequence Tag (EST), sequencing is an efficient means to generate functional genomic level data for non-model organisms or those with genome characteristics prohibitive to whole genome sequencing. EST sequencing is an attractive alternative to whole genome sequencing because the majority of most eukaryotic genomes is non-coding DNA, and EST sequences lack introns and intragenic regions that render analysis and interpretation of data more difficult [2]. ESTs thus have a high functional information content, and often correspond to genes with known or predicted functions [2,3]. Large collections of EST sequences have proven invaluable for gene annotation and discovery [2,4], comparative genomics [5], development of molecular markers [6,7], and for population genomic studies of genetic variation associated with adaptive traits [8]. Nonetheless, until recently, traditional laboratory methods for the development of EST resources have required costly and time consuming approaches involving cloning, cDNA library construction, and many labor intensive Sanger sequencing runs [2].

Massively parallel sequencing technologies, such as 454 pyrosequencing, remove many time consuming steps involved in Sanger sequencing of ESTs and have facilitated transcriptome sequencing at a fraction of the time and cost previously required [5,9-11]. At present, a single run on a 454 GS XLR70 Titanium pyrosequencer can produce more than 10^6 ^sequences averaging greater than 300 base pairs (bp) in length. The *de novo *assembly of the large numbers of short reads produced from this and similar technologies is a significant challenge for whole genome sequencing of large and complex genomes. In contrast, for transcriptome sequencing, *de novo *assembly is facilitated by the possibility of increased coverage depth (number of reads per nucleotide in the template) for the much smaller number of nucleotides in the transcriptome than in the whole genome [4]. In addition, the reduced amount of repetitive DNA found in genes compared to non-coding regions ameliorates one of the principal obstacles to *de novo *assembly of short reads [12]. Whereas most applications of parallel sequencing of ESTs have involved model organisms with draft genomes available to aid in assembly [4,13,14], recent studies have demonstrated highly successful *de novo *assemblies of 454 EST data for organisms with no prior genomic resources [5,7,15,16]. The generation of such large-scale sequence data will enable functional analyses that were previously limited to model organisms and their rapid application in ecologically important taxa [17]. Here, we utilize pyrosequencing of cDNA to characterize the transcriptome of lodgepole pine (*Pinus contorta*) and to develop genomic resources to support further research in this and other pines.

*P. contorta *is an ecologically and economically important tree that is widespread in the mountainous regions of western North America [18]. It is a fire-adapted species that mediates regeneration after disturbance, has a major impact on forest structure and ecology, and is a foundation species of many montane forest ecosystems. It is one of the most variable pines, and grows in a variety of conditions ranging from low elevations to timberline [19] where it has experienced and evolved in response to diverse selection pressures including that from variation in seed predator communities [20-23] and fire regime [24,25]. The current mountain pine bark beetle (*Dendroctonus ponderosae*) epidemic is causing unprecedented mortality of *P. contorta *throughout the Rocky Mountains [26], which is likely to cause rapid and massive changes in community structure and ecosystem processes. Consequently, a greater understanding of fine-scale population genetic variation and the genetic control of traits important to these forests would be beneficial and timely.

Although a large number of EST sequences for loblolly pine (*P. taeda*) exist in public databases (e.g., NCBI), far fewer resources exist for *P. contorta *(1 EST prior to 2010, as of January 2010 ca. 40,000 ESTs) and other pines, despite the importance of the genus. This paucity exists in part because pines have enormous genomes (10,000-40,000 mega-base pairs vs. 115 Mbp in *Arabidopsis thaliana*) with large amounts of repetitive DNA [27,28], making whole genome sequencing projects difficult or impractical. The construction of large EST collections is thus the most promising approach for providing functional genomic level information in pines [29]. Whereas other labs are currently generating *P. contorta *ESTs using Sanger sequencing (K. Ritland and J. Boehlman, pers. comm.), additional sequencing effort is needed to increase genomic level resources. The development of genomic resources for *P. contorta *should facilitate basic and applied research on the genetics and evolutionary ecology of this species and its role in maintaining forest health and ecosystem function [29,30]. In addition, EST collections for *P. contorta *will contribute to the development of molecular markers for other pines and facilitate comparative genomics and the study of adaptive variation across the genus.

Here we describe 454 pyrosequencing of *P. contorta *cDNA and assess the utility of this approach for transcriptome characterization and marker discovery in a species with a large and complex genome. Normalized cDNA collections from multiple tissues and individuals were used to sample large numbers of expressed genes and to detect simple sequence repeats (SSRs) and single nucleotide polymorphisms (SNPs). We first describe the assembly and functional annotation of EST sequences, and the level of transcriptome coverage provided by our sequence data. Second, we discuss the detection and characterization of a surprisingly large number of sequences representing retrotransposons. Finally, we utilize our assembled sequence data for the development of a variety of gene-based markers for population genomic studies, including SSRs occurring within regions that are conserved with another pine species, and SNPs occurring in regions with many reads and deep coverage. We designed high quality PCR primers for a large number of the SSRs we characterized, providing an immediately available resource of genetic markers for pines. Along with other recent studies [5,7,15,16], our results demonstrate the utility and highlight some of the challenges of next generation transcriptome sequencing applied to non-model organisms.

## Results and Discussion

### Results

#### 454 sequencing and assembly

**Figure 1:**
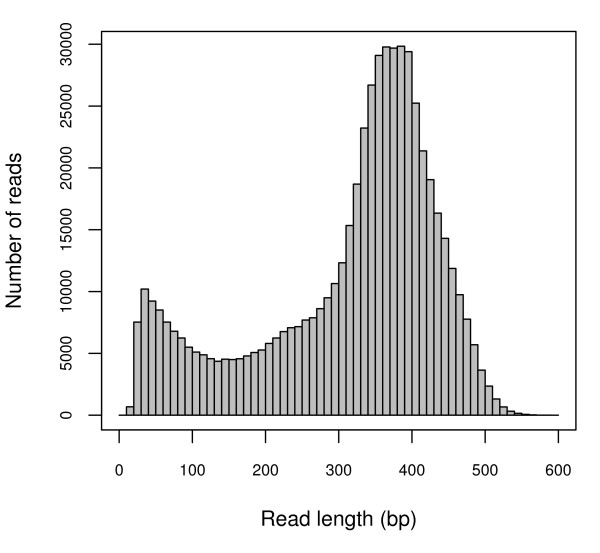
**Frequency distribution of 454 sequencing read lengths**. The frequency distribution of read lengths resulting from 454 GS XLR70 Titanium pyrosequencing.

We created a normalized cDNA pool based on RNA extracted from needles and developing conelets that were sampled from four individual *P. contorta *trees in the Medicine Bow National Forest in Wyoming. Pyrosequencing of this cDNA pool on a 454 GS XLR70 Titanium platform produced approximately 180 Mbp of sequence data, in the form of 586,732 reads averaging 306 bp in length (Fig. 1). Assuming a similar number of genes occur in *P. contorta *as in *A. thaliana *(25,000) and a similar average gene length of 2,000 bp [31], average transcriptome coverage was estimated at 3.6×. We removed a variety of reads with characteristics that could interfere with assembly prior to further analyses. After removing all reads that matched to three well-characterized conifer retrotransposons (11,394) [32-34], sequences containing long simple sequence repeats (3,681), and sequences not passing stringent quality screening (average quality score < 18; 106,761 reads), 464,896 reads entered assemblies run in Seqman Ngen (DNAstar, Inc.).

We first evaluated how varying some of the starting parameters affected *de novo *assembly of reads by running 18 different assemblies with different combinations of minimum match percentage, minimum match size, and gap penalty. Predictably, the number of assembled sequences and the length of assembled contigs increased with decreasing match percentage and decreasing match size (Table 1). However, the number of contigs was largest with a match percentage of 90%, suggesting that a substantial number of contigs (including paralogs) may be collapsed and joined when match percentages are dropped as low as 85% and a larger number of reads are assembled into a smaller number of contigs (Table 1). Analyses throughout this paper are based on a combination of reference-based and *de novo *assemblies. We first executed a reference-based assembly utilizing a set of approximately 19,000 unigenes available for *P. taeda *using a minimum match percentage of 88%, a minimum match size of 19 bp, and a gap penalty of 30. This assembly was executed to provide a set of contigs with sequences that are conserved between *P. taeda *and *P. contorta *and to use the comparative sequence alignments to design molecular markers with high cross-species transferability. *De novo *assembly of the remaining reads was run with a minimum match percentage of 93%, a minimum match size of 19 bp, and a gap penalty of 30. This combination of reference-based and *de novo *assemblies increased the number of assembled reads and the number of contigs when compared to pure *de novo *assembly (Table 1).

**Table 1:** Results and characteristics of assemblies run with different parameter settings.

		Gap Penalty			
		30		50	
		
Match %	Match length	Number of contigs and average length	Number of assembled reads	Number of contigs and average length	Number of assembled reads
85%	19	50,689 (537)	255,711	51,058 (533)	252,299
	23	49,621 (516)	248,537	50,039 (513)	245,563
	25	48,962 (507)	244,120	49,366 (504)	241,289

90%	19	55,886 (499)	220,517	55,996 (497)	217,169
	23	53,724 (490)	213,344	53,824 (488)	209,916
	25	52,770 (485)	209,735	52,726 (483)	206,167

95%	19	51,289 (443)	156,578	51,908 (441)	154,605
	23	46,611 (441)	142,428	46,585 (439)	140,071
	25	44,752 (439)	137,096	44,674 (437)	134,566

Combination of reference-based (match % = 88) and *de novo *assemblies (match % = 93)					
88%,93%	19	63,687 (500)	225,517		

Results from assemblies run in Seqman Ngen with different starting values for gap penalty, minimum match size, and mimimum match percentage. We present the number of contigs, average contig size (in parentheses), and the number of reads assembled into contigs for each different set of parameter settings.

**Figure 2:**
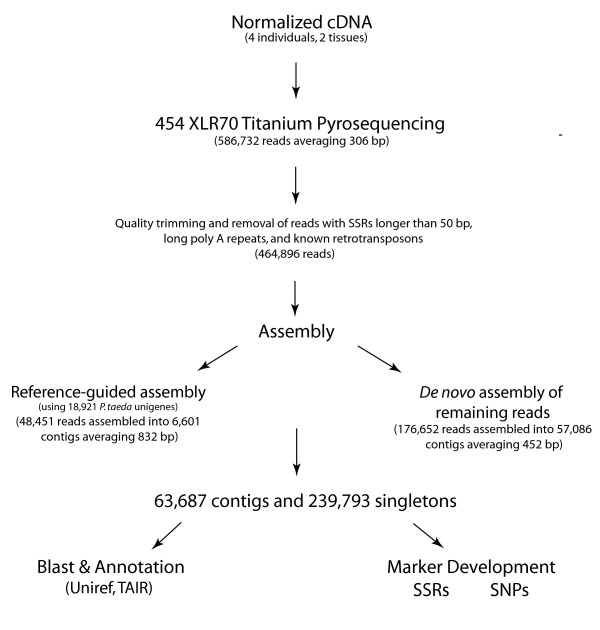
**Schematic of 454 EST analysis**. The steps and sets of sequences involved in 454 EST sequencing, assembly of reads into contigs, annotation using protein databases, and genetic marker discovery and characterization.

**Figure 3:**
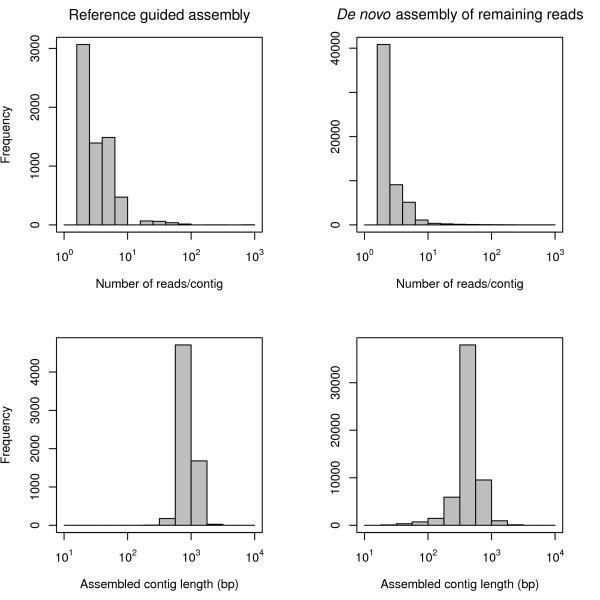
**Assembly characteristics of 454 ESTs**. Histograms depicting the number of reads per contig and contig lengths obtained from a reference-guided assembly (left) and a *de novo *assembly of the remaining reads (right).

**Figure 4:**
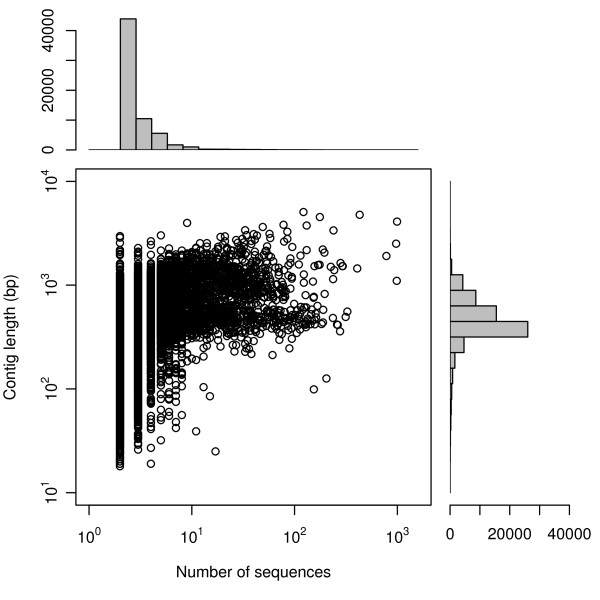
**Contig length as a function of the number of sequences assembled into each contig**. The marginal histograms depict the frequency distributions of the number of sequences assembled into contigs and the frequency distribution of contig length.

The reference-guided assembly placed 48,451 reads into 6,601 contigs (Fig. 2), which averaged 832 bp in length and had a mean coverage depth of 2.04 (Fig. 3; skewed distribution; 25% quantile, 1.3; 75% quantile, 2.02). *De novo *assembly of reads not assembled to the *P. taeda *unigenes placed 176,652 reads into 57,086 contigs with an average length of 452 bp and an average coverage depth of 2.1 (Fig. 3; 25% quantile, 1.4; and 75% quantile, 1.98). As expected, the length of contigs generally increased with the number of sequences assembled into them (Fig. 4). The combined assemblies had an average contig length of 500 bp and contained a substantial number of large contigs (Fig. 3). 7,828 contigs were larger than 800 bp in length, and the largest 10% were between 852 and 5,062 bp in length. 239,793 unassembled high quality reads were treated as singletons in further analyses (Fig. 2).

#### Annotation

To assess the coverage and quality of our assembly, we first used BLASTx [35] to align both contigs and singletons to the UniRef50 15.4 [36] and the TAIR9 *Arabidopsis thaliana *[37] annotated protein databases using an *E *value threshold of 10^-6^. Of 63,687 contigs, 20,301 (32%) had BLAST hits to known proteins in UniRef50 and matched 8,316 unique protein accessions (Table 2). As expected, a lower percentage of the shorter singleton reads had BLAST hits to UniRef50 proteins. Of 239,793 singleton reads, 30,836 (13%) had blast hits to UniRef50, with matches to 10,574 unique proteins (Table 2). Smaller numbers but similar percentages of contigs and singletons had BLAST hits to the TAIR database (Table 2). The majority of the annotated sequences corresponded to known plant proteins, with 9.8% matching conifer sequences (Table 3). In addition, a large number of sequences (10.4%) were most similar to fungal proteins (Table 3), likely indicating the presence of endophytic fungi in our sampled tissues. This seemingly low percentage of ESTs with BLAST hits is partially due to a high frequency of short sequences in our ESTs, although annotation of only 30-40% of sequences is common in analyses of large EST collections [5,16,38]. Longer contigs were more likely to have BLAST matches to the annotated protein databases; logistic regression indicated that EST sequence length was a significant predictor of the presence or absence of a significant BLAST match to one of the annotated protein databases (slope = 0.0058, intercept = -3.899, *P *< 0.0001). 85% of our contigs and singletons over 800 bp in length had BLAST matches, whereas only 5% of contigs and singletons shorter than 250 bp did. Nonetheless, BLAST searches identified a total of 17,321 unique protein accessions, indicating that our 454 sequencing project detected a substantial fraction of *P. contorta *genes.

**Figure 5:**
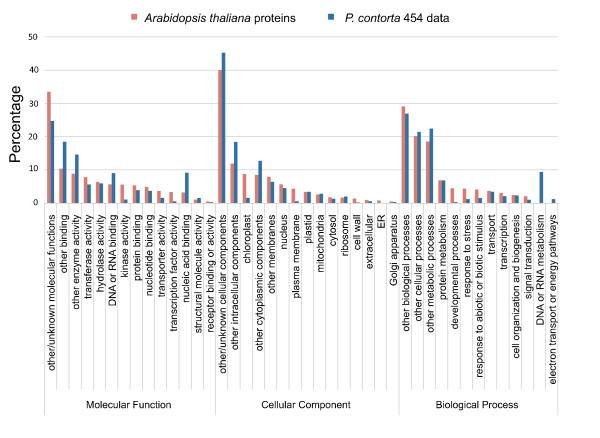
**Gene ontology assignments for P. contorta and A. thaliana**. Proportion of annotated contigs and singletons from *P. contorta *454 ESTs and annotated *A. thaliana *proteins that matched various gene ontology (GO) categories.

Using Blast2go (v.2.3.6; [39]), we were able to assign gene ontology classes to 4,890 (31%) of the 15,683 unique genes with BLAST matches to known proteins in Uniref50. There were a total of 21,351 gene ontology terms associated with these 4,890 unique genes. Of these, assignments to the molecular function ontology made up the majority (11,997, 56.2%) followed by biological process (5,400, 25.3%) and cellular components (3,954, 18.5%, Fig. 5). To compare the distribution of gene ontology annotations in our *P. contorta *454 data to that of the *A. thaliana *genome, the unique genes from both our data and the TAIR *A. thaliana *annotated database were mapped to respective TAIR GO Slim terms using Blast2go. GO Slim terms are a specified subset of higher-level ontology categories that provide a broad profile for genome-genome comparison [40]. The percentages of annotated *P. contorta *sequences assigned to GO Slim classes generally mirrored those of *A. thaliana *genes (http://www.arabidopsis.org; Fig. 5), reflecting a similar distribution of genes in different functional categories, and further highlighting that a large diversity of *P. contorta *transcripts is represented by these sequences.

**Table 2:** 454 EST matches to annotated protein databases.

	Contigs (63,687)		Singletons (239,793)		Combined Set (303,480)	
UniRef50						
Matches to database	20,301	(32%)	30,836	(13%)	51,137	(17%)
Unique matches to proteins	8,316	(13%)	10,732	(4%)	15,683	(5%)
						
TAIR9						
Matches to database	11,663	(18%)	12,799	(5%)	24,462	(8%)
Unique matches to proteins	5,635	(9%)	5,025	(2%)	8,385	(3%)

Numbers and percentages of 454 ESTs in the assembled contigs, singletons, and the combined sequence set with matches to known proteins in BLASTx searches of two annotated protein databases (Uniref50, [36]; TAIR9 [37]).

**Table 3:** Summary and taxonomic source of BLAST matches to 454 ESTs.

Taxonomic category	Contigs		Singletons		Combined Set	
Conifer	1,170	(14.1%)	966	(9.0%)	1,536	(9.8%)
Other plant	4,984	(59.9%)	5,191	(48.4%)	8,309	(53.0%)
Insect	363	(4.4%)	1,176	(11.0%)	1,413	(9.0%)
Fungi	388	(4.7%)	1,392	(13.0%)	1,625	(10.4%)
Protozoa	104	(11.3%)	172	(1.6%)	240	(1.5%)
Other Eukaryote	1,208	(14.5%)	1,614	(15.0%)	2,260	(14.4%)
Bacteria	82	(1.0%)	199	(1.9%)	270	(1.7%)
Virus	16	(0.2%)	22	(0.2%)	30	(0.2%)

Number and percentages of unique best BLASTx matches of 454 EST contigs, singletons, and the combined sequence set to Uniref50 grouped by taxonomic category.

**Figure 6:**
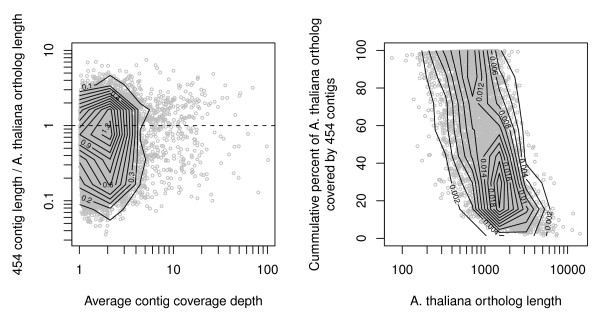
**Comparison of P. contorta contigs to orthologous A. thaliana coding sequences**. A. The ratio of *P. contorta *contig length to *A. thaliana *ortholog length as a function of contig coverage depth. The dotted line corresponds to a ratio of one, where 454 contigs are as long or longer than the BLAST matched *A. thaliana *orthologs. The contour lines on both plots correspond to the density of points in the plot. B. Total percent of *A. thaliana *ortholog coding sequence that was covered by all *P. contorta *contigs, as a function of the length of the *A. thaliana *coding sequence.

To assess the extent of transcript coverage provided by our 454 contigs and to evaluate how coverage depth affected the assembly of full length transcripts, we plotted the ratio of contig length to *A. thaliana *ortholog (from BLAST) length against coverage depth. Among the 454 contigs, there was a slight trend for increased coverage depth to result in higher coverage of the coding regions (*r*^2 ^= 0.02, *β *= 0.015, *P *< 0.0001), although a substantial number of deeply covered contigs fail to cover complete coding regions of their *A. thaliana *ortholog (Fig. 6A). Values greater than 1 in Fig. 6A for the ratio of 454 contig length to the length of *A. thaliana *ortholog coding region are likely due to UTR and other non-coding regions present in our contigs, but also indicate substantial coding region coverage by many individual 454 contigs. In many cases, multiple contigs covered different regions of *A. thaliana *orthologs. Plotting the summed proportion of *A. thaliana *orthologs covered by all 454 contigs reveals that a large number of orthologs are thoroughly covered by 454 contigs (Fig. 6B), although this coverage declines steeply with increasing ortholog length.

#### Assessment of retrotransposon abundance

BLAST searches of our 454 reads using a variety of criteria indicated an abundance of retrotransposon-like sequences in our data. Searches using queries of three known retrotransposons common in conifers (IFG7, GYMNY, and PpRT1; [32-34]) had matches to 11,394 sequences in our complete set of 454 reads. Additional searches of 17 known plant retrotransposon sequences against EST collections from ten plant taxa and our full set of raw 454 ESTs indicated that retrotransposons are present at a much higher frequency in our 454 ESTs than in other plant EST collections (Table 4). After BLAST annotation of our unique sequences, we further searched for terms identifying other proteins associated with retroelements. This resulted in an additional 3,485 unique sequences (representing a total of 13,325 reads) that were identified as proteins associated with retroelements. Together these three approaches indicate that as many as 36,325 (6.2%) of the raw 454 reads may represent transcriptionally active retroelements.

**Table 4:** Summary of plant ESTs with BLAST matches to retrotransposons.

Organism	# of ESTs	# of ESTs matching RTs	% Total
*Arabidopsis thaliana*	1,527,298	920	0.0602
*Triticum aestivum*	1,066,854	1,123	0.1053
*Vitis vinifera*	353,941	217	0.0613
*Populus nigra*	51,361	84	0.1635
*Populus tremula*	37,313	15	0.0402
*Populus trichocarpa*	89,943	94	0.1045
*Picea glauca*	297,913	210	0.0705
*Picea sitchensis*	168,675	54	0.0320
*Pinus pinaster*	27,847	15	0.0539
*Pinus taeda*	328,628	217	0.0660
***Pinus contorta***	**586,372**	**22,862**	**3.8989**

Number of ESTs matching to 17 well-characterized plant retrotransposon sequences in BLASTn searches of large EST collections for 10 selected plant taxa and the entire set of *P. contorta *454 reads.

In research on other genomes, small segments of retrotransposon sequences have been found within ESTs containing genes, which could contribute to the large number of BLAST searches matching to retrotransposon-like sequences [41]. To assess whether our sequences that aligned to *Pinus *retrotransposons IFG7, PpRT1, and GYMNY were located in coding regions, following [41] we assembled the corresponding sequences into 553 contigs using SeqMan Ngen. These contigs were then aligned to UniRef50 using BLASTx with an *E *value threshold of 10^-4^. If pieces of retroelement sequences were in genes, we would expect these contigs to align with known proteins in UniRef50 that were non-retroelement products [41]. Of the 553 query sequences, 315 contigs had hits to UniRef50 proteins, and these sequences matched to 105 unique protein accession numbers. These accession numbers were imported into the online UniProt database search tool (http://www.uniprot.org/jobs/; [40]) and manually inspected to assess whether the associated protein products belonged to retroelements or not. Of the 105 unique proteins, 19 proteins (18.1%) were not retroelement protein products determined by either protein name, known function, conserved motifs, or affiliated gene ontology terms. This suggests that the majority of the sequences that were similar to known retroelements are likely to reflect transcriptionally active retrotransposons, although a significant proportion (18%) of retrotransposon-like sequences may represent transcribed regions containing short pieces of retroelement-like sequences.

#### Marker identification and characterization

Using Perl scripts we identified a total of 15,084 di-, tri-, or tetra-nucleotide SSR regions with a minimum of four contiguous repeating units in contigs from the reference-based and *de novo *assemblies (Table 5). We identified 1,841 potential SSRs in contigs aligned to the *P. taeda *unigenes in the reference-based assembly (Table 5), and a much larger number of SSRs in the contigs built through the *de novo *assembly. Di-nucleotide repeats were by far the most common SSRs in our ESTs, with tri- and tetra-nucleotide repeats being present at much smaller frequencies (Table 5). 7,906 SSRs occurred in contigs with BLAST matches to Uniref50 annotated proteins, of which a substantial number (5,678) occurred in actual protein coding sequences. Although the majority of these contigs consisted of coding sequence, the density of SSRs was still higher in coding (0.0034 SSRs per bp) than non-coding regions (0.0018 SSRs per bp).

**Table 5:** SSRs in 454 ESTs.

Type of SSR repeat	Reference based assembly	*De novo *assembly of remaining reads	Total
Di	1,267 (54)	10,366 (1,487)	11,633 (1,541)
Tri	551 (520)	2,480 (1,748)	3,031 (2,268)
Tetra	23 (19)	397 (192)	420 (211)

Total	1,841 (593)	13,243 (3,427)	15,084 (4,020)

Numbers of di-, tri-, and tetranucleotide simple sequence repeats (SSRs) occurring in contigs from both the reference-based and *de novo *assemblies. In parentheses following the number of loci identified are the number of potentially amplifiable loci for which PCR primers were designed in BatchPrimer3 [42] with stringent criteria (see Additional file 1: Supplemental Table S1).

We were able to design high quality PCR primers with optimal expected product sizes and other properties that should facilitate successful amplification for a large portion these SSRs using BatchPrimer3 [42]. Primers were constructed for nearly a third of the SSRs identified, but quality priming sites occurred in a much higher proportion of tri- and tetra-nucleotide repeats than of di-nucleotide repeats (Table 5). Information on the name of the sequence containing each SSR, SSR motif, number of repeats, expected length of PCR product, sequences and positions of forward and reverse primers, and the GC content and melting temperature for each primer is included in Additional file 1: Supplemental Table S1. Of 96 loci tested for succesful PCR amplification in a single trial, 70% successfully amplified in *P. contorta *and 65% successfully amplified in *P. ponderosa*. In addition, more than 50% of the tested SSRs were polymorphic across panels of eight individuals of *P. contorta *and *P. ponderosa*.

Using Seqman Pro (DNASTAR, Inc.), we identified a substantial number of SNPs in our largest and most deeply covered contigs. The 20 BLAST-annotated contigs with the largest numbers of reads contained 301 SNPs with alternate alleles in at least 20% of reads and minimum coverage depth of 10×. These contigs represent 27,496 bp of sequence data, giving a SNP occurrence rate of 1.1 SNPs per 100 bp. Across all the contigs with more than 20 reads (658 contigs containing 614,125 nucleotides) we identified a total of 3,707 (0.6 high quality SNPs per 100 bp) high quality SNPs with frequency of the alternative allele being at least 20% and a minimum coverage depth of 8×.

## Discussion

Normalized cDNA collections from multiple tissues and individuals allowed us to sequence a large fraction of the *P. contorta *transcriptome using 454 pyrosequencing. The approximately 1.8 × 10^8 ^base pairs of data produced here represent a substantial sequence resource for *P. contorta *and will contribute to genomic data available for the genus *Pinus*. Below we discuss the characteristics and level of transcriptome coverage in our data, the abundance of transcriptionally active retrotransposons, the characterization of different molecular marker types, and future prospects for population genomics studies.

### Assembly and annotation

As with other recent studies [5,7,15,16], our results indicate that short reads from 454 sequencing runs can be effectively assembled and used to readily characterize the gene space of non-model organisms. The large number and length of reads generated from a half plate pyrosequencing run on a 454 GS XLR70 Titanium instrument resulted in a relatively high expected average transcriptome coverage depth (3.6×). As expected with the increased read lengths from the 454 GS XLR70 Titanium instrument, our contigs were on average larger (mean = 500 bp) than those assembled in previous studies that used earlier 454 technologies with shorter reads (e.g., 197 bp, [5]; 247 bp, [7]; 440 bp, [16]). In addition, a large percentage of our contigs were greater than 800 bp in length and had good coverage depth (Figs. 3, 4). High quality of assembled contigs was indicated by a high proportion of contigs matching to known proteins using BLAST searches and by the ready PCR amplification of SSR markers developed in contigs.

Nonetheless, a relatively small portion (48%) of reads were assembled into contigs, which is significantly less than that reported for several other recent 454 transcriptome assemblies (e.g., 91%, [5]; 88%, [7]; 90%, [16]). As a consequence, coverage depth and contig length was lower than expected for many contigs. Large numbers of singletons could result from a variety of causes, including the assembly algorithm used, artifacts of cDNA normalization, genes expressed at low levels, contaminants from other organisms such as bacterial or fungal parasites, or 454 sequencing errors [12]. Transcriptome assembly is additionally hindered by alternative splicing, among other issues [5,17]. Long repeat regions, which are particularly common in pines, are known to cause difficulties with *de novo *assembly [12,43]. One factor potentially influencing difficulty in assembly is the abundance of both simple and complex repeats. Although we removed a substantial number of reads prior to assembly, both long SSR regions and retroelements were prevalent in our data and could have contributed to difficulty with assembly. Additional filtering steps could improve the fraction of reads assembling into contigs [44], although this could also involve an overall loss of information. Nonetheless, many singletons were high quality reads and matched to proteins in BLAST searches, highlighting that they are still a very important source of information. Given that next generation sequencing projects will soon become common in non-model organisms, increases in the amount and quality of data should result in the improvement of *de novo *assembly algorithms over time [17].

Estimating the number of genes and the level of transcript coverage represented in an EST collection is an important issue for transcriptome sequencing projects, but is difficult or impossible without a completely annotated reference genome sequence. We indirectly evaluated transcriptome coverage breadth by first determing the number of unique genes detected in our sequence collection using BLAST. The large number of sequences that matched in BLAST searches to unique proteins (Table 2) indicates that our 454 sequencing reads identified a substantial portion of the genes in *P. contorta*. A large portion of the 17,321 unique genes had best BLAST hits to plant proteins, with a smaller portion hitting to other taxonomic groups (Table 3). A similarly large number of these were assigned to a wide range of gene ontology categories (Fig. 5), indicating that a wide diversity of transcripts are represented by our sequence data. Furthermore, many of the contigs and singletons without BLAST hits likely represent additional genes not represented in the annotated protein databases we searched, or genes that lack BLAST matches due to short length. If we assume a similar number of genes occur in pines as in *Arabidopsis *(25,000, [31]), our annotated sequences are likely to represent more than half of the genes in *P. contorta*. To evaluate the extent to which individual transcripts were fully covered, we quantified coverage of full length *A. thaliana *orthologs by our 454 contigs. A substantial number of *A. thaliana *orthologs were entirely covered by 454 contigs, with increasingly complete coverage for contigs with higher coverage depths (Fig. 6A). Similarly, the completeness of ortholog coverage decreased with decreasing contig coverage depth and increasing length of the *A. thaliana *sequence, indicating that additional sequencing would be warranted for more comprehensive transcriptome coverage (Fig. 6A, B).

ESTs with BLAST hits to distant taxa may represent proteins not well-characterized in closely related taxa, but could also represent RNA from other organisms present in sampled tissues. The substantial number of BLAST hits to known fungal proteins in our ESTs (Table 3) is most likely due to the presence of fungi in the needles and or developing conelets we sampled. This is not surprising given the prevalence of endophytic and symbiotic fungi in conifer tissues [45,46]. Similarly, [5] reported matches in BLAST searches of their 454 ESTs to a substantial number of non-metazoan proteins, and identified the presence of an important intracellular parasite known to affect butterfly population dynamics. Such findings point to the utility of large-scale transcriptome sequencing and annotation for detecting parasites or symbionts present in sampled in tissues, just as is done more generally in environmental genomics [47].

### Transcriptionally active retrotransposons

Retrotransposons are known to play a prominent role in the evolution of genome size and complexity across eukaryotes [48]. For example 30-50% of the mammalian genome is composed of retroelements [9], and they comprise a substantial proportion of most plant genomes [49,50]. In addition, the proliferation of retrotransposons may be a major factor contributing to the large genome sizes of pines, where retrotransposons may account for almost 2% of the genome [34]. Although they are typically not actively transcribed into RNA, their presence in EST collections indicates that they can be transcriptionally active, and in some cases at very high levels (Table 4; [51,52]). BLAST searches of known retrotransposon sequences in plants as well as results from BLAST annotation of our contigs and singletons indicate that more than 6% of our 454 reads represent retrotransposon-like sequences. They are present in our data at a level an order of magnitude higher than in comparable EST collections for other plants, and two orders of magnitude higher than in other conifers (Table 4). We recognize that a variety of issues may contribute to this result, including possible variability of cDNA normalization across studies or tissues. Nonetheless this result highlights the abundance of retrotransposons that may occur in large-scale EST sequencing projects and deserves attention for several reasons. First, repetitive DNA is a known difficulty for assembly of short sequences into contigs, and the prevalence of retrotransposon sequences could contribute to assembly difficulties. Consequently, the characterization of retrotransposons in our 454 ESTs will be useful for planning future transcriptome sequencing in this species, where it may be desirable to use laboratory methods to remove retroelements from the cDNA sequencing pool and enrich for non-repetitive templates. Finally, given that a major goal of our study was molecular marker development, identification of the retrotransposon-derived reads is crucial to avoid developing and genotyping molecular markers residing in such sequences.

### Marker identification and characterization

Polymorphic genetic markers are important for research involving population genetic structuring, demography, relatedness, and the genetic basis of adaptive traits [53-55]. Next generation transcriptome sequencing leads to superior resources for the development of such markers not only because of the enormous amount of sequence data in which markers can be identified, but also because discovered markers are gene-based. Such markers are advantageous because they facilitate the detection of functional variation and the signature of selection in genomic scans or association genetic studies [2,56]. Currently few genetic marker resources exist for *P. contorta *(but see [57]). The large number of SSRs and SNPs we detected provide a wealth of markers potentially useful to applications ranging from population genetics, linkage mapping, and comparative genomics, to gene-based association studies aimed at understanding the genetic control of adaptive traits.

EST-based SSRs are advantageous compared to SSRs located in non-transcribed regions owing to their higher amplification rates and cross-species transferability [58]. We have identified large numbers of SSRs in our 454 EST collection, and were able to design high quality PCR primers for nearly a third of these regions (Table 5). Moreover, a large number of these SSRs occur in the protein coding sequences of annotated contigs representing genes of known or predicted identity and function. That most of these SSRs occur in coding sequences (5,678 out of 7,906) and not UTRs is perhaps surprising. Another possibly surprising result is that the majority of SSRs in the coding regions were di-nucleotide repeats (78.4% di-, 19.4% tri-, and 2.1% tetra-nucleotide repeats). These di-nucleotide SSRs were typically short (< 20 bp), and coding regions with di-nucleotide repeats almost always contained more than one SSR, although these were not always in close proximity where they could be considered interrupted repeats.

We were also able to design primers for a large subset of the SSRs located in regions where *P. contorta *ESTs were aligned to *P. taeda *ESTs (Table 5). As priming sites should be highly conserved, these markers are likely to be transferable to related species, and may represent a valuable source of genetic markers for the genus *Pinus*. We were able to successfully amplify a large percentage of polymorphic SSR loci in initial tests with *P. contorta *and *P. ponderosa*, validating the quality of our assembled contigs and the utility of the SSRs produced. Our ongoing work is assessing amplification rates, polymorphism levels and cross-species transferability for hundreds of these SSRs across multiple species of pines. The density of SSRs detected in 454 sequencing projects depends on numerous factors including the template sequenced and the criteria used in recognizing SSRs, and has varied greatly across recent studies [5,59,60]. Nonetheless, these studies and our own highlight the value of 454 sequencing as a cost and time effective route for rapid SSR discovery.

Because of the deep and redundant coverage produced over many genes, pyrosequencing of cDNA is ideal for SNP discovery and characterization [5,6,16]. Although our sampling was limited to four different individuals, high quality SNPs are abundant in our contigs. In addition, most of these SNPs reside in annotated genes, which will allow the identification of reading frame and facilitate more detailed analyses on the significance of molecular variation. The SNP frequency in our contigs (0.6/100 bp) is in the general range of that reported in other studies using 454 pyrosequencing of cDNA pooled from multiple individuals (e.g., [6] 0.33/100 bp [5] 0.67/100 bp, [16] 0.49/100 bp, [61] 0.72/100 bp). Studies such as these highlight the value of next generation sequencing projects for SNP discovery and characterization. Similar to studies on *P. taeda *[62,63] and *Picea glauca *[8], the availability of large numbers of SNPs should facilitate population genomic and gene-based association studies in *P. contorta*.

## Conclusions

Pines are among the most ecologically and economically important plant species on Earth. Yet, the size and complexity of their genomes has hindered the development of genomic resources for many taxa in the genus. The 303,480 unique sequences in this 454 EST collection represent a major genomic level resource for *P. contorta*, and will be useful for comparative genomic studies in pines. This highlights the utility of high-throughput transcriptome sequencing as a fast and cost-effective road to rapidly obtain information on coding genetic variation in pines. Because of large and minimally structured populations, high levels of nucleotide diversity, and rapid decay of linkage disequilibrium, conifers represent excellent subjects for association genetic studies [27,64]. Nonetheless, the enormous size of the pine genome means that millions of markers will be required for the fine-scale mapping of traits. Consequently, utilizing SNPs occurring in candidate genes or in ESTs is a promising avenue for association genetics in pines [27,64]. Until recently, association genetic studies for adaptive variation have been limited in conifers (but see [62,65]) due to the high cost and time required by traditional routes for the development of marker resources. The ability to investigate the genetic basis of adaptive traits in these trees should increase as groups working on pine genomic resources continue to build large EST data sets through next generation sequencing approaches. The thousands of SNP and SSR markers in our 454 ESTs should enable population genomic and gene-based association studies [53,66]. Such analyses should contribute to understanding patterns of adaptive variation across the genome and to identifying the genetic basis of adaptive traits.

## Methods

### Sequencing and assembly

Fresh needles and developing conelets were sampled from four individual *P. contorta *trees in the Medicine Bow National Forest of south central Wyoming (USA). RNA extraction, cDNA synthesis, and 454 sequencing were performed by staff at the biotechnology company GATC (GATC, Inc.). Total RNA was isolated using a CTAB-based protocol and was further purified with the NucleoSpin RNA XS kit (Macherey and Nagel, Inc.). RNA was pooled from the four individuals for cDNA synthesis. From this pool, poly(A)+ RNA was prepared, and first-strand cDNA synthesis was primed with an N6 randomized primer. 454 adapters A and B (Roche Life Sciences, Inc.) were ligated to the 5' and 3' ends of the cDNA, and the cDNA was PCR-amplified using a proof reading enzyme. One cycle of denaturation and reassociation of the cDNA was used to obtain N1 cDNA. Single stranded cDNA was used for hybridization rather than double stranded cDNA, and normalization of the cDNA template was achieved by separating reassociated double stranded cDNA from the single stranded cDNA by passing the mixture over a hydroxylapatite column [15,67]. Following hydroxylapatite chromotography, single stranded cDNA was PCR amplified. One *μ*g of cDNA was sequenced in two half-plate runs on a 454 GS XLR70 Titanium genomic sequencer (Roche, Inc.). Although technical difficulties with the first run resulted in a smaller than expected number of reads, these reads were included in analyses as they were still of high quality. Files containing the sequences and quality scores have been deposited at NCBIs Short Read Archive (accession SRA012089). 454 primer sequences were trimmed from all reads prior to assembly. Because repetitive DNA is a known problem for the assembly of short pyrosequencing reads, and repeats are abundant in conifer genomes, we removed a large number of reads from the full data set to avoid sequences that could interfere with assembly. We discarded reads with simple sequence repeats longer than 50 bp, and a substantial number of reads matching to three well-characterized conifer retrotransposon sequences (see below). We also removed reads with average quality scores less than 18. We used Seqman Ngen (DNAstar, Inc.) to assemble reads into contigs, as this program has been successful in assembling 454 sequences from transcriptomes [5,68,69] and other programs created to assemble next generation sequencing data often assemble few EST reads into contigs [14,17]. The assembly algorithm employed by Ngen is an extension of work by [70] that allows the incorporation of quality scores for individual nucleotides, and can be specifically parameterized for assembling short pyrosequencing reads. Sequences are placed into a hash table of overlapping subsequences, before sequential assembly of overlapping reads occurs with near constant memory usage (for a detailed explanation of the algorithm see [71]).

To explore the effects of parameter settings on the outcome of assemblies, we ran *de novo *assemblies with a range of minimum match percentages (85%, 90% and 95%), match lengths (19, 23, and 25 bp), and gap penalties (30 and 50) (Table 1). Analyses presented in this paper are based on a combination and reference-based and *de novo *assemblies. We first executed a reference-based assembly using a set of 18,921 unigenes constructed from *P. taeda *Sanger ESTs http://www.ncbi.nlm.nih.gov/UniGene/UGOrg.cgi?TAXID=3352. For this assembly, we used a minimum match size of 19 nucleotides, match percentage of 88%, mismatch penalty of 18, and gap penalty of 30. We used a lower minimum match percentage here because of substantial divergence between taxa. We utilized this approach not only to facilitate assembly but also to produce a set of contigs containing sequences conserved between *P. contorta *and *P. taeda*. These served as a basis for characterizing molecular markers likely to have high cross-species transferability. Reads that did not join into contigs in the reference-based assembly were entered into a *de novo *assembly with a more stringent minimum match percentage. This assembly was run with a minimum match size of 19 nucleotides, match percentage of 93%, mismatch penalty of 18, and gap penalty of 30. The resulting contigs and remaining singletons were then combined into a single set (Fig. 2).

### Annotation

To assess the coverage and quality of our assembly, we first used local BLASTx [35] to align both contigs and singletons to the UniRef50 15.4 [36] and the TAIR9 *Arabidopsis thaliana *[37] annotated protein databases using an *E *value threshold of 10^-6^. BLASTx results were passed through a custom Perl pipeline that summarized information and produced tab-delimited tables with accession numbers, gene name, taxonomic ID, query length, ortholog sequence length, sequence alignment, *E *value, and bit score for each protein accession with matches in BLAST searches. To estimate the proportion of annotated contigs and singletons that matched to unique genes in the two databases, these files were then filtered for redundancy in protein accessions. Assignment of gene ontology (GO) terms to ESTs with BLASTx matches was then performed by importing the accession numbers for the BLASTx hits to unique proteins into Blast2go (version 2.3.6; http://www.blast2go.org/). Blast2go is an automated tool for the assignment of gene ontology terms to BLAST hits and was designed for use with novel sequence data [39]. We also generated gene ontology assignments for *A. thaliana *annotated proteins to compare the distribution of functional annotations in *P. contorta *to that from a plant with a well-characterized transcriptome.

### Assessment of retrotransposon abundance

Because preliminary analyses identified a very large number of retrotransposon like sequences in our data, we quantified the presence of retroelements in the raw 454 data and the set of contigs and singletons with BLASTn searches using two approaches. First, we used BLASTn to search for 17 known plant retrotransposon sequences in our entire set of 454 reads and in large EST collections from other selected plant taxa. These retrotransposon sequences represent complete sequences from the NCBI Nucleotide database http://www.ncbi.nlm.nih.gov/entrez?db=nuccore that match queries of 'copia-like' and 'gypsy-like' in plant taxa. Additionally, complete retrotransposon sequences from conifer genomes were selected, including IFG7, PpRT1, and Gymny [32-34]. Ten species were chosen for comparison of retrotransposon abundance on the basis of taxonomic relatedness to our study species and the number of ESTs available. Entire EST collections available in dbEST for these species, as well as our complete set of *P. contorta *454 reads, were compared to the 17 retrotransposon sequences using BLASTn with an *E *value threshold of 10^-6^. Second, following BLAST annotation of contigs and singletons, we further searched for sequences representing proteins associated with retroelements by identifying sequences that had the terms "copia", "gag", "pol", "retroelement", "integrase", "reverse transcriptase" and "retrotransposon" in their annotation.

### Marker identification and characterization

We wrote Perl programs to identify SSRs in our contigs and to identify a subset of these that reside in contigs where reads from *P. contorta *were assembled onto *P. taeda *unigenes. We located di-, tri-, and tetra-nucleotide SSRs with lengths less than 50 bp and with a minimum of 4 contiguous repeating units, which provided a large number of candidate SSRs. We determined which SSRs occurred in coding sequences of genes by extracting the aligned portions of sequences having BLAST matches to annotated protein coding orthologs, and then using the same algorithm as above to detect SSRs in both the aligned and remaining portions of these contigs. The design of high quality PCR primers is crucial for the development of molecular markers that can be readily usable, and suitable priming sites will not exist for all of the loci we identified. To determine the loci that represent good candidates for PCR amplification, we used the program BatchPrimer3 [42] to construct PCR primers in the flanking regions of SSRs. This program identifies SSRs, allows the control of many parameters to facilitate high quality primer construction, and can simultaneously process thousands of sequences. We designed primers in the flanking regions of SSRs that were a minimum of 12 bp long and used stringent criteria to design genetic markers of desired PCR product length and with a high probability of amplification. We created primers with a minimum GC content of 30%, with a melting temperature between 52 and 62°C and a maximum 4°C difference between primers, and positioned primers to obtain PCR products between 100 and 450 bp long. We also constrained primer construction so that the end of each primer contained a GC clamp (the last two nucleotides were G or C). Additional settings used for primer design are available from the authors upon request.

We tested 96 of these primer sets for succesful SSR amplification in several individuals in *P. contorta *and *P. ponderosa*. PCR reactions consisted of 50-100 ng total genomic DNA; 2 pmol of each primer; 0.5 mM each of dATP, dCTP, dGTP, and dTTP; 1× PCR buffer; and 0.4 units of taq polymerase. All polymerase chain reaction amplifications were performed with the following conditions; 94°C for five minutes, followed by 32 cycles of 94°C for one minute, 50°C for one minute, and 72°C for one minute, followed by a final extension step of 72°C for three minutes. We ran 12 *μ*l of each product out on 1.5% agarose gels stained with ethidium bromide, and scored each individual for successful amplification. A subset of SSR loci that amplified successfully were tagged with flourescently labelled M13 tails, run out on an ABI 3130 genetic analyzer (ABI, Inc.), and genotyped for polymorphism.

We also identified an abundance of SNPs in large contigs with high coverage depths using the SNP reporter feature in Seqman Pro (DNAstar, Inc.). We considered only SNPs with nucleotide variation, and disregarded indels. First, we enumerated and visually inspected high quality SNPs with coverage depth of at least 10× and with an alternate allele in a minimum of 20% of the reads for the 20 contigs containing the largest number of reads. Second, we assessed the presence of high quality SNPs in all contigs containing greater than 25 reads. Here we counted SNPs at sites where coverage depth was at least 8×, and where alternate alleles were present at a minimum frequency of 20%.

## Authors' contributions

TLP organized and planned the research, contributed to all aspects of analysis, and drafted the manuscript. KSG wrote Perl scripts, contributed to analyses involving BLAST and retrotransposon sequences and contributed to manuscript preparation. JAG provided programming expertise, wrote bioinformatics scripts, and guided analyses involving BLAST and SSR characterization. CWB contributed to conceptual planning of the research, provided funds, and contributed to manuscript preparation. CAB provided funding, computational guidance, and was substantially involved in research design, data analysis, and manuscript preparation. All authors have read and approved the final manuscript.

## Supplementary Material

Additional file 1 **Supplementary File S1 -- Primer sequences for SSR loci**. Information for SSR primers designed in BatchPrimer3 using stringent criteria. Provided for each locus is information on the sequence ID, location of SSR, location of both primers, primer sequences, length of primer sequences, melting temps, GC%, SSR motif, motif length, SSR sequence, and SSR length. Click here for file
